# Successful Transition From Topical Ophthalmic Drops to Cream Formulation in the Management of Mild Allergic Conjunctivitis: A Case Report

**DOI:** 10.7759/cureus.79818

**Published:** 2025-02-28

**Authors:** Mai Nishio, Eisuke Shimizu, Kazuki Asai, Kazumi Fukagawa, Hiroshi Fujishima

**Affiliations:** 1 Department of Ophthalmology, Takenotsuka Aoba Eye Clinic, Tokyo, JPN; 2 Department of Research, Yokohama Keiai Eye Clinic, Yokohama, JPN; 3 Department of Research, OUI Inc., Tokyo, JPN; 4 Department of Ophthalmology, Keio University School of Medicine, Tokyo, JPN; 5 Department of Ophthalmology, Ryogoku Eye Clinic, Tokyo, JPN; 6 Department of Ophthalmology, Tsurumi University, Yokohama, JPN

**Keywords:** allergic conjunctivitis, allergy treatment, cream, epinastine, itchy

## Abstract

This case report highlights the successful transition from epinastine eye drops to a novel epinastine eyelid cream in managing mild allergic conjunctivitis. A 33-year-old Japanese female with mild allergic conjunctivitis, previously managed with 0.1% epinastine hydrochloride eye drops, requested an alternative formulation due to daily contact lens wear. Treatment was changed to a once-daily application of 0.5% epinastine hydrochloride eyelid cream. Clinical assessment and symptom evaluation were performed at baseline and after 28 days using slit-lamp examination and the Japanese Allergic Conjunctival Disease Quality of Life Questionnaire. Following the transition, the patient's clinical signs (mild conjunctival hyperemia and moderate papillae) and subjective symptoms (itching and mild discomfort) remained stable. Slit-lamp examination showed no corneal or limbal pathology, and tear fluid immunoglobulin E (IgE) testing remained positive, consistent with mild allergic conjunctivitis. No adverse effects or cutaneous reactions were observed. This case suggests that epinastine eyelid cream may serve as an effective once-daily alternative to conventional eye drops in mild allergic conjunctivitis. While this represents the first documented successful transition between delivery methods, larger clinical trials are warranted to confirm these findings and explore applications across various presentations and severities of allergic ocular disease.

## Introduction

Allergic conjunctivitis is a common ocular condition characterized by a type 1 hypersensitivity reaction, resulting in inflammation of the ocular surface, itching, tearing, and photophobia [1,2]. It is primarily mediated by immunoglobulin E (IgE)-induced mast cell activation, which triggers histamine release and subsequent inflammation [3]. In addition, Th17 cells and interleukin-17 (IL-17) contribute to the Th2-driven pathogenesis, amplifying immune responses in allergic conjunctivitis [4].

The condition can be classified into acute forms, such as seasonal and perennial allergic conjunctivitis, and chronic forms, including vernal keratoconjunctivitis, atopic keratoconjunctivitis, and giant papillary conjunctivitis [2].

Conventional treatments often include topical ophthalmic drops containing antihistamines, mast cell stabilizers, or dual-action agents, which provide short-term symptom relief by inhibiting histamine activity and preventing mast cell degranulation [5,6]. These medications are effective in reducing symptoms such as itching, redness, and tearing and are commonly used in combination with nonsteroidal anti-inflammatory drugs, corticosteroids, or immunosuppressants, depending on disease severity [6-8].

Epinastine, a dual H1-receptor antagonist and mast cell stabilizer, has been primarily available in the form of ophthalmic eye drops for managing allergic conjunctivitis symptoms. It blocks H1 histamine receptors to reduce itching and redness while stabilizing mast cells to prevent the release of inflammatory mediators [1]. Recent advancements have explored alternative delivery methods, such as topical eyelid creams, which provide a non-invasive and patient-friendly option. A phase 3 clinical trial in Japan demonstrated that a 0.5% epinastine eyelid cream significantly reduced ocular itching and conjunctival hyperemia with a favorable safety profile and enhanced patient satisfaction [1].

This case reports a patient with mild allergic conjunctivitis who successfully transitioned from topical ophthalmic drops to a cream formulation, highlighting the potential benefits of this novel treatment approach.

## Case presentation

A 33-year-old Japanese female, who presented with mild ocular pruritus, successfully transitioned from epinastine eye drops to eyelid cream formulation for allergic conjunctivitis management. Her medical and family histories were unremarkable. The patient was a long-term soft contact lens wearer (>12 hours daily) and had been managing symptoms with twice-daily 0.1% epinastine hydrochloride ophthalmic solution (ALESION LX Ophthalmic Solution; Santen Pharmaceutical Co., Ltd., Osaka, Japan).

For the clinical examination, initial visual acuity (VA) with the right eye (RV) = 0.2 (1.2 × S - 1.50D C - 1.50D Ax 90), left eye (LV) = 0.15 (1.2 × S - 0.50D C - 1.50D Ax 90) where S indicates sphere, D indicates diopter, C indicates cylinder, and Ax indicates axis. Intraocular pressures (IOP) were measured at 17.0 mmHg in the RV and 13.0 mmHg in the LV.

The Japanese Allergic Conjunctival Disease Quality of Life Questionnaire (JACQLQ) scores revealed the following [9]: a symptom score of 9 out of 40 (cutoff value ≥ 8), a daily living score of 7 out of 68 (cutoff value ≥ 6), and a social functioning score of 2 out of 4 (cutoff value ≥2).

Objective clinical signs were scored according to the Japanese Guidelines for Allergic Conjunctival Diseases 2020 [10], which evaluate the following parameters using slit-lamp microscopes (700GL, TAKAGI, Nagano, Japan; Smart Eye Camera, OUI Inc., Tokyo, Japan) [11-13]. Palpebral conjunctiva: hyperemia (none-severe), swelling (none-severe), follicles (none-severe), papillae (none-severe), giant papillae (none-severe). Bulbar conjunctiva: hyperemia (none-severe), chemosis (none-severe). Limbus: swelling (none-severe), Horner-Trantas dots (none-severe). Cornea: epithelial disorder (none-severe).

**Figure 1:**
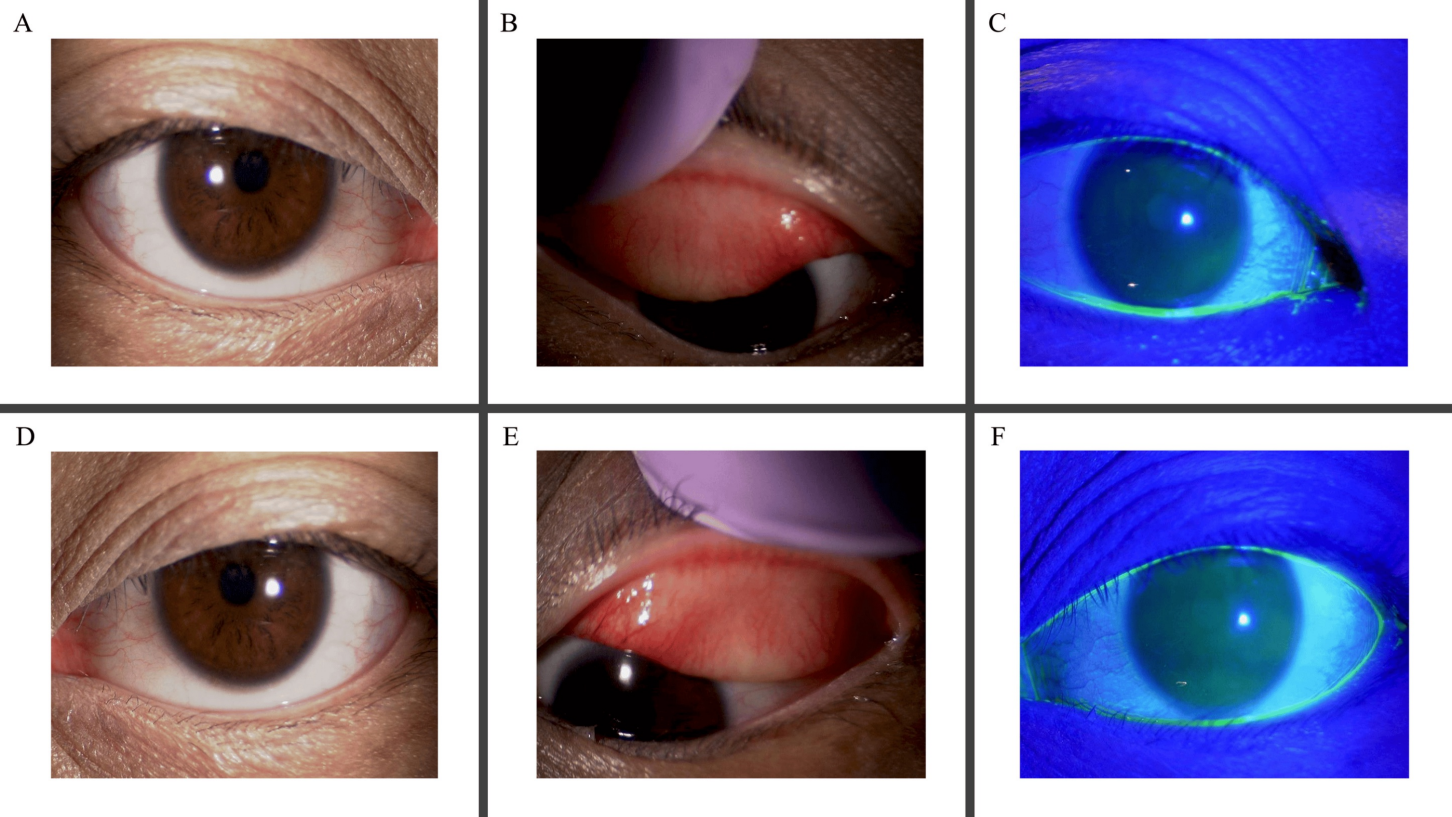
Clinical anterior segment images. Slit‐lamp images of the patient’s right eye (A-C) and left eye (D-F). (A, D) Mild to moderate hyperemia of the bulbar conjunctiva without chemosis. (B, E) Eversion of the upper eyelid shows palpebral conjunctiva with mild hyperemia, moderate papillae, no swelling, and no follicles; no giant papillae are noted. (C, F) Under cobalt blue illumination with fluorescein, the cornea displays no epithelial abnormalities. No limbal swelling or Horner‐Trantas dots were observed.

The patient exhibited the following findings (Figure 1). The palpebral conjunctiva shows mild hyperemia with no swelling or follicles, moderate papillae, and no giant papillae. The bulbar conjunctiva exhibits moderate hyperemia without chemosis. The limbus has no swelling or Horner-Trantas dots, and the cornea presents no epithelial disorder.

**Figure 2:**
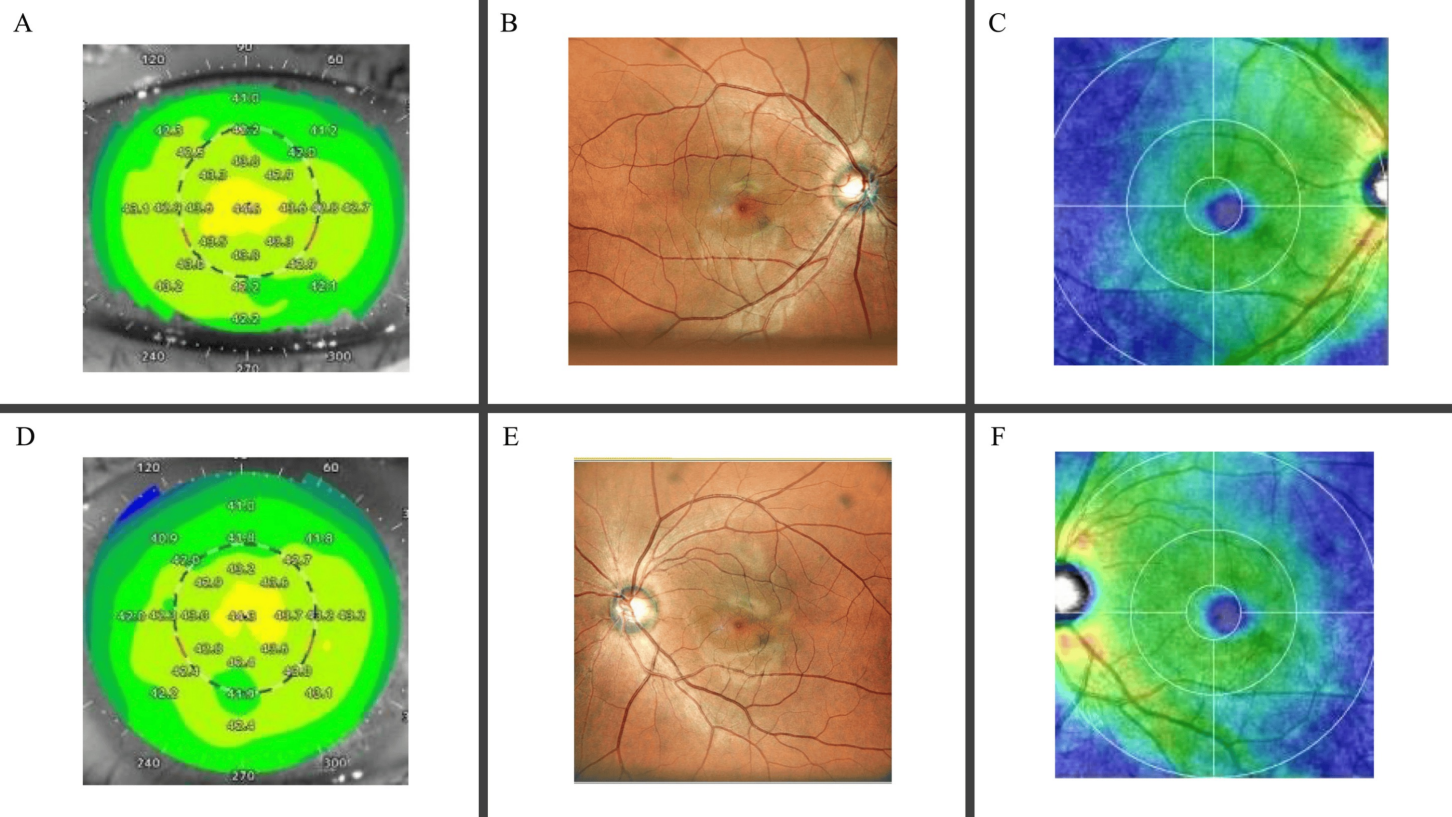
Corneal topography, fundus photography, and fundus optical coherence tomography analysis. Examination results of the right eye (A-C) and the left eye (D-F). (A, D) Corneal topography showing normal curvature and no abnormalities. The corneal curvature in the right eye measured 43.64 D at axis 176° and 42.94 D at axis 86°, while the left eye measured 43.42 D at axis 174° and 42.91 D at axis 84°. (B, E) Fundus photographs revealing an unremarkable retina and optic nerve disc. (C, F) Retinal ganglion cell complex thickness maps demonstrating no notable findings. No other remarkable abnormalities were observed.

There were no remarkable abnormalities observed in the crystalline lens, with no evidence of cataract. IOP were measured at 16.0 mmHg in the RV and 14.0 mmHg in the LV. Additionally, the vitreous, retina, and optic nerve appeared unremarkable, with no notable findings (Figure 2).

A rapid tear fluid IgE test (Aller Watch; Wakamoto Pharmaceutical Co., Ltd., Tokyo, Japan) confirmed positive IgE levels (total IgE concentration ≥10 IU/ml).

Laboratory testing for specific IgE antibodies (View 39 (allergy test that measures specific IgE antibodies); BML Inc., Tokyo, Japan) revealed sensitization to house dust, house dust mites, Japanese cedar (Sugi), and Japanese cypress (Hinoki), with all allergens classified as class 2 (range: 0-6; minimum 0, maximum 6).

The patient reported a desire to switch pharmaceutical formulations due to the inconvenience of using topical eye drops while wearing contact lenses. As there was no possibility of pregnancy, we transitioned the treatment from topical epinastine hydrochloride eye drops to a cream formulation (0.5% epinastine hydrochloride, ALESION Eyelid Cream; Santen Pharmaceutical Co., Ltd., Osaka, Japan), applied once daily. The patient adhered to the new regimen without issue. The cream formulation used per application is approximately 30 mg per eye, which corresponds to a cream length of about 1.3 cm when dispensed from the tube.

A follow-up evaluation was conducted 28 days later.

VA was recorded as follows: RV = (1.2 × S - 1.25D; C - 2.25D, Ax 90), LV = (1.2 × S - 0.50D; C - 1.75D, Ax 95) where S indicates sphere, D indicates diopter, C indicates cylinder, and Ax indicates axis. IOP was measured at 15.0 mmHg in the RV and 17.0 mmHg in the LV.

Clinical signs and subjective assessments remained largely unchanged. The JACQLQ scores were part I (symptoms): 8, part II (daily living): 8, part III (social functioning): 2.

**Figure 3:**
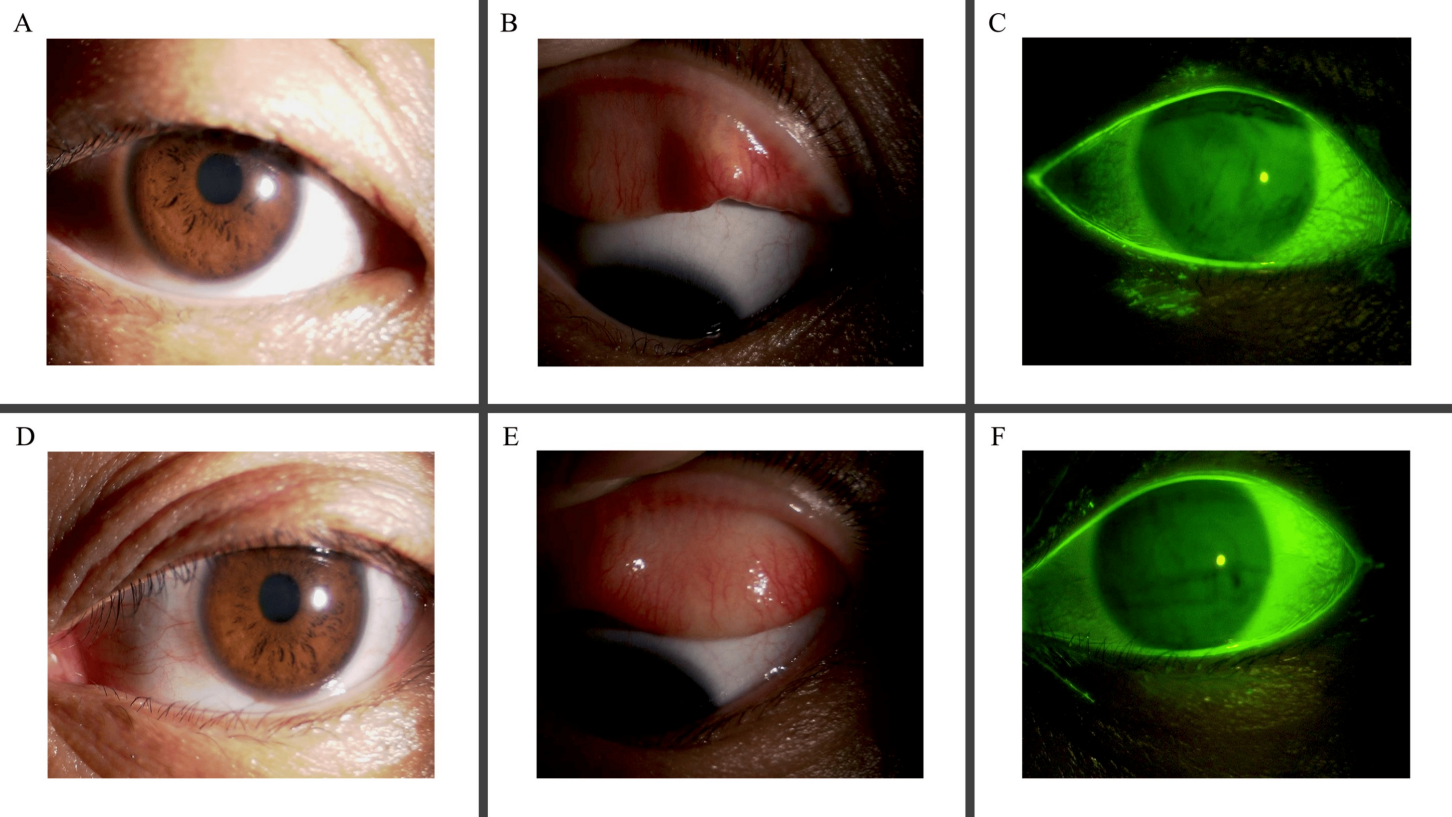
Clinical anterior segment images after 28 days of the treatment. Slit‐lamp images of the patient’s right eye (A-C) and left eye (D-F). (A, D) Mild hyperemia of the bulbar conjunctiva, with no chemosis. (B, E) On eyelid eversion, the palpebral conjunctiva shows mild hyperemia without swelling or follicles, moderate papillae, and no giant papillae. (C, F) Under fluorescein staining, the cornea appears free of epithelial disorders. No limbal swelling or Horner‐Trantas dots were observed.

Objective clinical signs scored according to the Japanese Guidelines for Allergic Conjunctival Diseases 2020 also remained stable (Figure 3).

The findings included mild hyperemia of the palpebral conjunctiva with no swelling or follicles, moderate papillae, and no giant papillae. The bulbar conjunctiva exhibited mild hyperemia without chemosis. The limbus showed no swelling or Horner-Trantas dots, and the cornea had no epithelial disorder.

Other clinical findings included no abnormalities in the eyelid or crystalline lens, with no evidence of cataract. The vitreous, retina, and optic nerve appeared unremarkable, consistent with previous evaluations. There were no signs of conditions reported in the past, such as blepharochalasis, blepharitis, or petechial keratitis.

Tear fluid IgE levels continued to be positive. The patient reported no adverse effects, including erythema, eye irritation, foreign body sensation, photophobia, eye pain, or tearing, all of which had been documented in prior reports. Additionally, no new discomfort associated with the cream formulation was observed. Based on these findings, the patient was advised to continue using the cream.

## Discussion

Our case highlights the successful transition from topical eye drops to a cream formulation for managing mild allergic conjunctivitis in a young Japanese female. Despite the change in treatment modality, the patient’s subjective symptoms and objective clinical findings remained stable, underscoring the potential utility of eyelid cream as an alternative to traditional eye drops. Furthermore, given the sustained efficacy observed in this case, it is worth considering whether eyelid cream could also be effective as a primary treatment modality rather than solely as a substitution. Future studies are warranted to explore its standalone therapeutic potential [1].

Epinastine cream has shown in vivo efficacy in type I allergic models, demonstrating its ability to permeate eyelid skin and deliver sustained therapeutic effects [14]. In rabbit studies, the gradual transdermal absorption of epinastine applied to the eyelid was observed, supporting its potency as a persistent, skin-penetrating agent [15]. At a concentration of 0.5%, it effectively suppresses type I allergic reactions, making it a promising once-daily treatment for allergic conjunctivitis [15].

Previous studies have established that epinastine 0.05% eye drops provide symptom relief for up to 4-8 hours, improving ocular itching and conjunctival hyperemia. The new 0.5% epinastine cream formulation leverages transdermal absorption through the eyelid skin, extending the duration of action to approximately 24 hours [1]. This innovative method simplifies application by targeting the outer skin of the upper and lower eyelids, ensuring effective delivery to the conjunctiva via transdermal absorption [15].

In the context of glaucoma management, strategies to improve compliance include the use of smart eye drop bottles, automated reminders, and patient education [16]. However, cream formulations offer a novel and user-friendly alternative, potentially enhancing patient adherence through ease of application [1]. By bypassing challenges associated with conventional eye drop use, such as discomfort, overflow, or improper technique, eyelid creams could ensure reliable drug delivery even for elderly patients who struggle with eye drop application and often misplace drops on their cheeks instead of their eyes. Additionally, for children who dislike eye drops, this formulation allows medication to reach the conjunctiva while keeping their eyes closed, making administration easier and more tolerable. Furthermore, for patients requiring multiple eye drops, maintaining the appropriate interval between administrations can be a barrier to adherence. By using an eyelid cream formulation, this issue can be mitigated, simplifying the treatment regimen and potentially improving adherence. Thus, eyelid creams could address common barriers to adherence [1].

A prior study has indicated the potential of epinastine eyelid cream in managing vernal keratoconjunctivitis, particularly in a child's age [17]. However, further investigation is needed to evaluate its use in pediatric populations, as current evidence is predominantly based on adult data [17].

The prophylactic use of anti-allergic eye drops, which are essential in the treatment of allergic conjunctivitis, before the onset of itching has been shown to improve quality of life. Therefore, a formulation requiring less frequent administration would be advantageous [18].

To our knowledge, this is the first documented case demonstrating a transition from eye drops to an eyelid cream without compromising therapeutic outcomes. Furthermore, this represents the first reported case worldwide of an adult patient using epinastine cream as a substitute for eye drops, highlighting its potential as an alternative treatment modality for allergic conjunctivitis. While these findings are promising, larger studies with diverse patient cohorts are required to confirm these results and explore applications across various allergic conjunctivitis subtypes.

## Conclusions

Epinastine hydrochloride cream represents a promising alternative for managing allergic conjunctivitis, particularly for patients seeking simplicity and improved adherence. Ongoing research and clinical evaluations will further clarify its role in routine ophthalmologic practice.
